# Treatment of Isolated Idiopathic Growth Hormone Deficiency in Children and Thyroid Function: Is the Need for LT4 Supplementation a Concern in Long-Term Therapy?

**DOI:** 10.7759/cureus.21722

**Published:** 2022-01-30

**Authors:** Daniela Salazar, Vicente Rey, João Sergio Neves, César Esteves, Rita Santos Silva, Sofia Ferreira, Carla Costa, Davide Carvalho, Cintia Castro-Correia

**Affiliations:** 1 Endocrinology, Diabetes and Metabolism, Centro Hospitalar Universitário São João, Porto, PRT; 2 Pediatrics, Centro Hospitalar São João, Porto, PRT; 3 Endocrinology, Diabetes and Metabolism, Centro Hospitalar São João, Porto, PRT; 4 Endocrinology, Hospital da Luz, Porto, PRT; 5 Pediatric Endocrinology and Diabetology Unit, Centro Hospitalar Universitário São João, Porto, PRT; 6 Pediatrics, Centro Hospitalar Universitário São João, Porto, PRT

**Keywords:** thyroid function, gh deficiency, children, hypothyroidism, somatropin

## Abstract

Introduction

Recombinant human growth hormone (rhGH) replacement therapy might be able to induce hypothyroidism, but this is a controversial issue. Previous studies evaluated the effects of rhGH replacement therapy on thyroid function, but little information is available in the subset of children with isolated idiopathic growth hormone deficiency (GHD). Our aim was to assess the effects of rhGH replacement therapy on thyroid function in children with isolated idiopathic GHD.

Methods

Retrospective analysis of the medical files of 64 children with confirmed GHD treated with rhGH. After review, 56 children with isolated idiopathic GHD and treated with rhGH for at least one year were included. Auxological (weight standard deviation score [SDS], height SDS, growth velocity [GV] SDS) and biochemical (free thyroxine [FT4], thyroid-stimulating hormone [TSH], and insulin-like growth factor 1 [IGF-1]) parameters were recorded before, during, and after treatment with rhGH.

Results

FT4 and TSH levels decreased significantly during rhGH therapy in children with isolated idiopathic GHD. Twenty-one percent (n=12) of the children developed hypothyroidism, on average 47 months after initiation of rhGH. Higher baseline FT4 levels were protective against the need for levothyroxine (LT4) (OR=0.8, CI 0.592-0.983; p=0.036). Hypothyroidism was reversed after interruption of rhGH, except in one patient; FT4 levels returned to baseline in the first year after completing the treatment. Final height SDS of the children who developed hypothyroidism was not different from their counterparts without hypothyroidism (-1.24 [-1.52 to -1.10] vs -1.13 [-1.78 to -0.74], p=1.000). Predicted adult height (PAH) SDS in patients who completed rhGH treatment was similar in both LT4 supplemented (n=7; final Ht SDS -1.16 [-1.31 to -1.10] vs PAH -1.00 [-1.42 to -0.48]; p=0.398) and not supplemented patients (n=25; final Ht SDS -1.46 [-1.83 to -0.78] vs PAH SDS -0.88 [-1.35 to -0.56]; p=0.074).

Conclusions

Our results show that patients with isolated idiopathic GHD may transiently need LT4 during GH treatment. Properly supplemented patients achieved PAH.

## Introduction

The incidence of growth hormone deficiency (GHD) in children has been estimated to be approximately 1:4000 to 1:10,000, probably underestimated in developing countries. Patients with proven GHD should be treated with recombinant human growth hormone (rhGH), in order to normalize height during childhood and to achieve normal adult height [1].

The impact of rhGH administration on thyroid function is a matter of controversy, as there is a complex relationship between the GH system and hypothalamus-pituitary-thyroid (HPT) axis [2]. Several studies in patients treated with GH showed an impact on thyroid hormones, and conflicting results show both unaltered [3, 4] or decreased serum thyroxine (T4) levels [2, 5-8], and unaltered [3-5, 8], increased [2, 7], and decreased [6] serum triiodothyronine (T3) levels. Most authors believe that GH induces increased peripheral T4 deiodination, with subsequent increase in T3 and decrease in serum reverse T3 (rT3) [9, 10], claiming an effect that is independent of thyroid-stimulating hormone (TSH). Other reports state that thyroid hormone changes are in fact the result of unmasking of previous unrecognized central hypothyroidism [11], due to inclusion of heterogeneous groups of patients in studies, some of whom may have hypopituitarism [6, 12]. And yet, others suggest that the effect of negative feedback on the pituitary gland is due to increased T3 production [13]. Moreover, differences in the composition of the pituitary GH formulations, that in some cases contained TSH and prolactin (PRL), and in hormone assay methods, were of concern in the past [2, 12].

The clinical significance of changes in the thyroid function during GH replacement therapy remains controversial. Nevertheless, it is known that hypothyroidism has deleterious effects on growth velocity (GV), and normal thyroid hormone secretion or appropriate substitution with levothyroxine (LT4) is necessary for the optimal effect of both endogenous GH and rhGH substitution on the growth velocity [14]. Some studies in children and adults reported the need for LT4 supplementation during rhGH therapy, in a variable percentage of patients who were euthyroid before rhGH therapy [15-18], but mainly in patients with multiple pituitary hormone deficiencies (MPHD) [12]. However, because follow-up reported in the literature is relatively short (usually up to 12 months) [6, 15, 19, 20], little information is available on the incidence of clinically relevant hypothyroidism during prolonged rhGH therapy.

Furthermore, anticipating which patients are more likely to develop hypothyroidism during rhGH therapy was addressed by few previous studies, particularly in the pediatric population. Considering that isolated idiopathic GHD is the most common cause of GHD in children and adolescents [21], and that these patients require long-term treatment with rhGH, it is important to improve our knowledge on the field.

Our aims were a) to evaluate the impact of rhGH therapy on thyroid function of euthyroid children and adolescents with isolated idiopathic GHD, and b) to explore which patients develop hypothyroidism during long-term rhGH treatment.

## Materials and methods

Study design and participants

We performed a retrospective study involving children referred for evaluation by Pediatric Endocrinology in a reference center for short stature or growth failure. Inclusion criteria were a confirmed diagnosis of GHD and treated with rhGH for at least one year. Selected patients started GH therapy between January 2004 and June 2019.

Children with organic brain lesions, history of head trauma or cranial irradiation, systemic diseases, or syndromes that result in growth disorders were excluded. Patients with MPHD, identified genetic defects causing GHD, and patients treated with LT4 prior to the start of GH due to primary or central hypothyroidism were also excluded.

GH deficiency was defined based on auxological criteria (GV standard deviation score [SDS] ≤ 0 and/or height SDS ≤ -2 for age and gender), bone age (BA) delay (over two years) compared to chronological age, and biochemical criteria (GH peak levels < 7 ng/mL in two provocative tests, clonidine and glucagon). All patients underwent nutritional assessment and laboratory testing to rule out alternative causes of short stature.

Auxological (weight [Wt], height, body mass index [BMI], and GV) and biochemical (free T4 [FT4], TSH, insulin-like growth factor 1 [IGF-1], and peak GH levels on provocative testing) parameters were recorded before, at 3-6 months of treatment with rhGH, and every six months thereafter, until 24-30 months of therapy. Height was measured using a Harpenden Stadiometer. Pubertal staging was evaluated according to the criteria set by Tanner and Whitehouse [22]. BA was assessed before rhGH and every 12 months, using the Greulich-Pyle atlas [23]. rhGH was administered once daily as a subcutaneous injection, at bedtime, and dosage was adjusted to obtain normal IGF-1 levels for pubertal state and sex.

Wt, height, BMI, GV, predicted adult height (PAH) and final height were expressed as SDS according to the 2007 World Health Organization (WHO) Growth Charts [24], as there are no national growth charts in Portugal. IGF-I SDS for age and sex was calculated.

Hypothyroidism was defined by the medical team as FT4 levels inferior to reference range for age in two consecutive measures, three to six months apart. LT4 dose requirement during follow-up was also recorded.

The study was conducted after obtaining the approval from the Ethics Committee, in accordance with the Declaration of Helsinki.

Serum analysis

Plasma TSH, FT4, IGF-1 and somatotropin (hGH) concentrations were measured by the chemiluminescent immunometric assay (CMIA). TSH and FT4 were assessed by Architect® i1000SR analyzer (Abbott, Abbott Park, IL, USA), and IGF-I and hGH by Immulite® 2000 Third Generation (Siemens, Munich, Germany). Normal range for TSH was 0.35 μIU/mL to 4.94 μIU/mL, with analytical sensitivity ≤ 0.0025 μIU/ml, and a 20% inter-assay coefficient of variance (CV) at <0.02 μIU/mL. The analytical range for FT4 was 0.7-1.48 ng/dl, with an analytical sensitivity of ≤0.4 ng/dL. For IGF-I, WHO NIBSC 1st IRR87/518 standard was applied, with analytical sensitivity 20 ng/ml, calibration range up to 1600 ng/ml, and intra-assay CV 3.1-5.8%. For hGH, normal range was 0.05-40 ng/mL (0.15-120 mIU/L), with analytical sensitivity 0.01 ng/mL (0.03 mIU/L), and intra-assay CV 5.3-6.5%.

Statistical analysis

Continuous variables are described as mean ± standard deviation or median (25th-75th percentiles) and categorical variables as proportions (percentages). Normal distribution was checked using Shapiro-Wilk test, or skewness and kurtosis. Comparison between baseline and treatment values of the same parameter was performed by paired t-test and Wilcoxon test. Comparison between groups (according to the need of LT4) was performed with t-test, U-Mann-Whitney, and Fisher’s test, as appropriate. Simple linear and logistic regression, and simple and multivariate cox regression were used to evaluate determinants of hypothyroidism and its occurrence. Statistical analyses were performed with SPSS software version 26.0 (IBM Corp., Armonk, NY). A two-sided p-value < 0.05 was considered statistically significant.

## Results

Medical files from 64 children with isolated GHD were reviewed. All children performed pituitary magnetic resonance imaging (MRI) and patients with changes on the image were excluded (ectopic pituitary, n=6; pituitary stalk interruption, n=2). Fifty-six patients with isolated idiopathic GHD were included in the study. All girls had a normal karyotype (46, XX), and all patients were euthyroid before starting rhGH.

Clinical and auxological features of children at baseline are shown in Table 1. A total of 57.1% (n=32) of the children were girls, with the mean age of 9.5 ± 3.26 years, Wt SDS of -1.8 ± 1.13, height SDS -2.6 [-3.07 to 2.35], BMI SDS -0.1 ± 1.14, and GV SDS of -2.1 ± 1.85. The mean lower GH peak at provocative testing was 3.3 ± 1.87 ng/mL. All children had birth weight appropriate for gestational age, and 80.0% (n=28 in 35) of patients with recorded information were prepubertal. rhGH treatment was given at a dose of 0.021 to 0.04 mg/kg/day (mean 0.032 ± 0.004 mg/kg/day).

**Table 1:** Characteristics of children with IIGHD before starting rhGH treatment (n=56). Values are shown as mean ± standard deviation or as median [95% confidence interval]. BMI – body mass index; FT4 – free thyroxine; GH – growth hormone; IIGHD – isolated idiopathic growth hormone deficiency; IGF-1 – insulin-like growth factor-1; PAH – predicted adult height; SDS – standard deviation scores; TSH – thyroid-stimulating hormone.

Characteristics		
Feminine sex (%)		57.1%
Age (years)		9.5 ± 3.26
Weight SDS		-1.8 ± 1.13
Height SDS		-2.6 [-3.07 to 2.35]
BMI SDS		-0.1 ± 1.14
Growth velocitySDS		-2.1 ± 1.85
PAHSDS		-0.9 ± 0.63
IGF-1 SDS		-1.1 ± 1.24
FT4 (ng/dL)		1.1 ± 0.13
TSH (µU/L)		2.6 ± 1.05
GH lower peak (ng/mL)		3.3 ± 1.87

Initiation of rhGH therapy had a significant impact in increasing Wt SDS, height SDS, GV SDS, and IGF-1 SDS, both at one and two years of follow-up, as presented in Table 2.

**Table 2:** Comparison of clinical and auxological parameters before and during rhGH treatment. Values are shown as mean ± standard deviation or as median [95% confidence interval]. BMI – body mass index; GV – growth velocity; IGF-1 – insulin-like growth factor-1; SDS – standard deviation scores; rhGH – recombinant human growth hormone. *vs pre-therapy values

	n	12-18 months therapy	p*	n	24-30 months therapy	p*
Weight SDS	36	-1.3 ± 1.16	<0.001	17	-0.9 ± 1.55	0.049
Height SDS	54	-2.0 [-2.53 to -1.31]	<0.001	24	-1.47 [-1.94 to -1.01]	<0.001
BMI SDS	54	-0.3 ± 0.97	0.109	24	0.015 ± 1.17	0.621
GVSDS	52	2.1 ± 2.71	<0.001	23	2.2 ± 4.06	0.004

By 12-18 months of therapy, 5.4% (n=3) patients had started LT4 therapy for new-onset hypothyroidism. A total of 12 patients (21.4%) needed supplementation with LT4 during follow-up (until May 2020), but only four started it in the first two years of rhGH therapy. Mean dose of LT4 was 0.7 (0.58-1.44) µg/kg/day. It took a mean of 46.9 ± 38.0 months (about four years) until the urge of hypothyroidism.

Time to LT4 start was associated with FT4 levels prior to rhGH therapy (HR=0.001; 95% CI 0.00-0.23; p=0.014), even after adjustment for patient’s age (HR=0.001; 95% CI 0.00-0.25; p=0.016) (Table 3).

**Table 3:** Association between baseline clinical, auxological and biochemical parameters and time to hypothyroidism onset (n=56). BMI – body mass index; FT4 – free thyroxine; GH – growth hormone; GV – growth velocity; HR – hazard ratio; IGF-1 – insulin-like growth factor-1; PAH – predicted adult height; SDS – standard deviation scores; TSH – thyroid-stimulating hormone; rhGH – recombinant human growth hormone. *data adjusted for age; **rhGH dose in mg/kg/day was multiplied by a factor of 100.

	Univariate analysis		Multivariate analysis*	
	HR [95% CI]	p	HR [95% CI]	p
Age, years	0.99 [0.82, 1.20]	0.920	-	-
Feminine sex	0.80 [0.24, 2.69]	0.720	0.80 [0.24, 2.69]	0.719
Puberty	0.88 [0.33, 2.32]	0.797	0.89 [0.35, 2.72]	0.813
Weight SDS	1.41 [0.69, 2.88]	0.347	1.56 [0.76, 3.23]	0.226
Height SDS	1.23 [0.66, 2.31]	0.513	1.29 [0.66, 2.51]	0.452
BMI SDS	1.07 [0.59, 1.95]	0.821	1.08 [0.59, 1.98]	0.803
GVSDS	1.04 [0.71, 1.52]	0.841	1.04 [0.71, 1.54]	0.831
IGF-1 SDS	1.11 [0.72, 1.70]	0.613	1.13 [0.72, 1.77]	0.598
FT4 (ng/dL)	0.001 [0.00, 0.23]	0.014	0.001 [0.00, 0.25]	0.016
TSH (µU/L)	1.12 [0.65, 1.91]	0.687	1.11 [0.65, 1.92]	0.701
GH lower peak (ng/mL)	1.06 [0.74, 1.53]	0.748	1.07 [0.72, 1.56]	0.732
rhGH dose**	0.19 [0.03, 1.25]	0.084	0.19 [0.03, 1.28]	0.090

**Figure 1:**
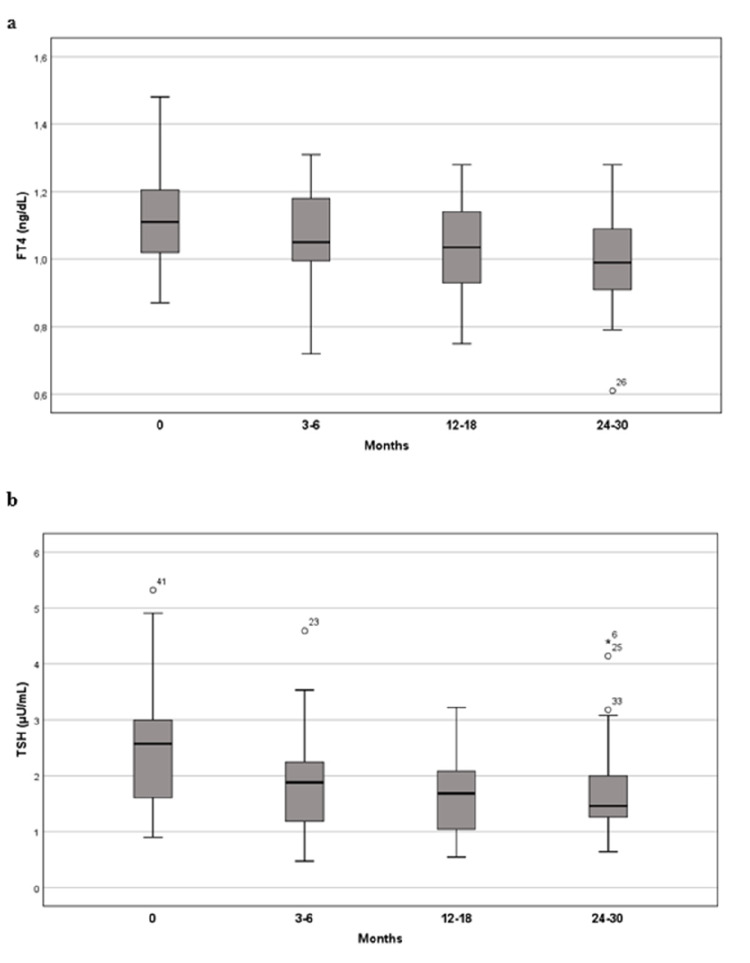
Boxplot of serum FT4 levels (a) and TSH levels (b) before and during rhGH treatment (n=52). FT4 – free thyroxine; TSH – thyroid-stimulating hormone; rhGH – recombinant human growth hormone.

**Figure 2:**
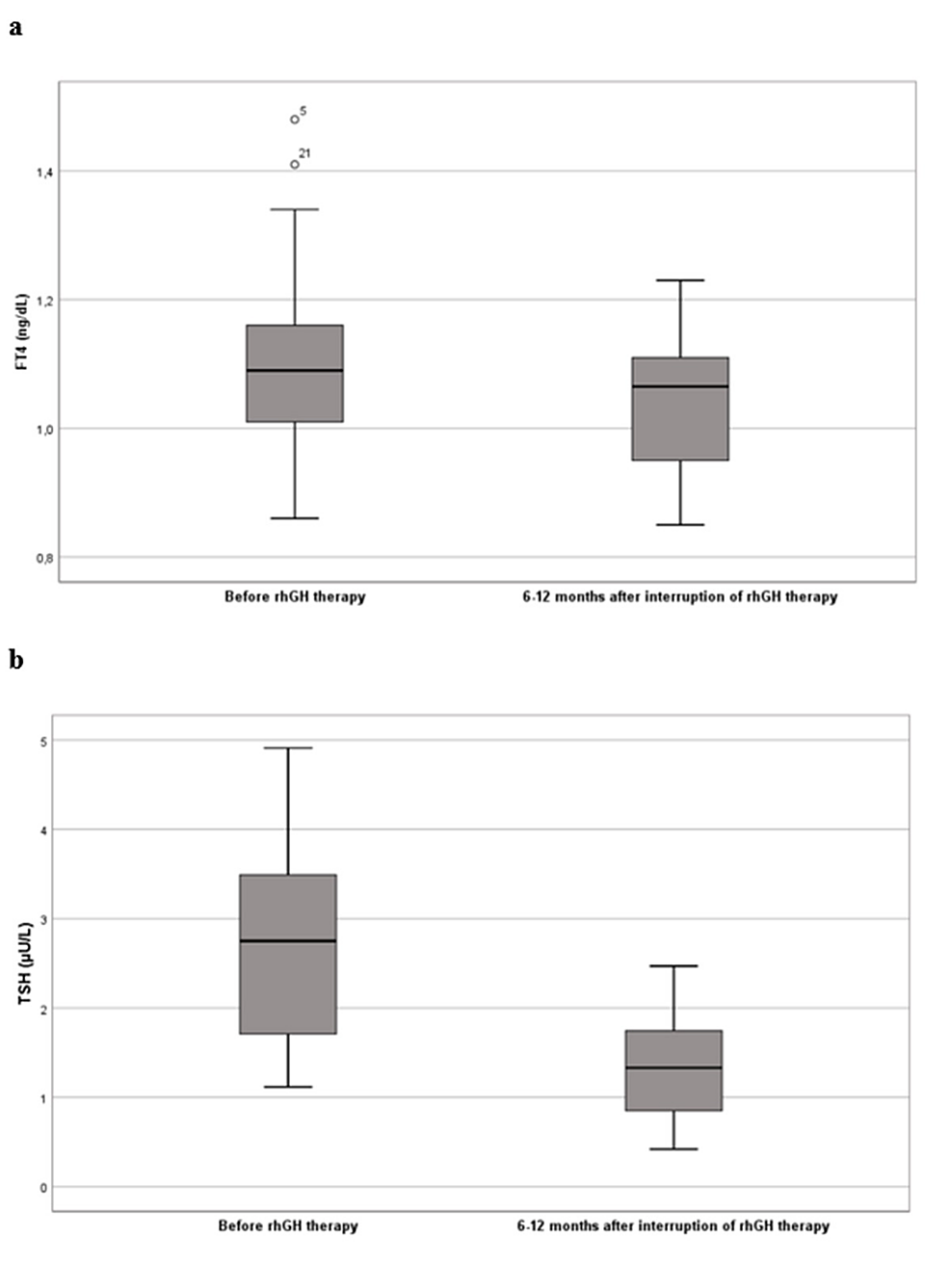
Boxplot of serum FT4 levels (a) and TSH levels (b) before initiating and 6-12 months after interruption of rhGH treatment (n=34). TSH – thyroid-stimulating hormone; FT4 – free thyroxine; rhGH – recombinant human growth hormone.

Table 4 and Figure 1 resume the evolution of thyroid function during the first 24 to 30 months of rhGH therapy (excluding patients who were started on LT4 at this point). A significant reduction in FT4 and TSH levels is seen since the first months of therapy and maintained over time. Six to twelve months after completing rhGH therapy (n=35), only one patient needed to maintain treatment with LT4; the remaining patients showed FT4 levels practically overlapping pretreatment levels (1.1 ± 0.40 vs 1.0 ± 0.12 ng/dL, p=0.041). TSH levels remained lower comparing with pre-treatment values (2.7 ± 1.10 vs 1.4 ± 0.58 µU/L, p<0.001), especially when looking at the group of patients not treated with LT4 (Table 5 and Figure 2).

**Table 4:** Comparison of thyroid function of patients before and during rhGH treatment (n=52). FT4 – free thyroxine; TSH – thyroid-stimulating hormone; rhGH – recombinant human growth hormone. *vs pre-therapy values

	Before therapy	3-6 months therapy	p*	12-18 months therapy	p*	24-30 months therapy	p*
FT4 (ng/dL)	1.1 ± 0.40	1.0 ± 0.12	0.005	1.0 ± 0.14	<0.001	1.0 ± 0.15	0.001
TSH (µU/L)	2.6 ± 1.06	1.9 ± 0.87	<0.001	1.7 ± 0.66	<0.001	1.7 ± 0.88	<0.001

**Table 5:** Comparison of thyroid function of patients before and after rhGH treatment in patients not treated with LT4 (n=34). FT4 – free thyroxine; LT4 – levothyroxine; TSH – thyroid-stimulating hormone; rhGH – recombinant human growth hormone. *vs pre-therapy values

	Before therapy	6-12 months after therapy	p*	LT4 supplementation (n=6)	p*	No LT4 supplementation (n=28)	p*
FT4 (ng/dL)	1.1 ± 0.38	1.0 ± 0.12	0.041	1.0 ± 0.11	0.306	1.0 ± 0.12	0.086
TSH (µU/L)	2.7 ± 1.10	1.4 ± 0.58	<0.001	1.7 ± 0.55	0.010	1.2 ± 0.56	<0.001

Comparing patients who required LT4 supplementation (n=12) during rhGH treatment with those who did not (n=44), we found no differences in pre-rhGH clinical and auxological parameters, except for a slightly higher rhGH dose in non-supplemented patients (0.030 ± 0.002 vs 0.032 ± 0.004 mg/kg/day; p=0.042), and lower baseline FT4 levels in the first group (1.0 ± 0.07 vs 1.1 ± 0.14 ng/dL; p=0.002) (Table 6). Adjusting FT4 levels for patient’s age, this association remains significant (OR=0.763, CI 0.592-0.983; p=0.036).

**Table 6:** Comparison of clinical, auxological and biochemical parameters before rhGH treatment according to LT4 supplementation (n=56). BMI – body mass index; FT4 – free thyroxine; GH – growth hormone; GV – growth velocity; IGF-1 – insulin-like growth factor-1; LT4 – levothyroxine; PAH – predicted adult height; SDS – standard deviation scores; TSH – thyroid-stimulating hormone; rhGH – recombinant human growth hormone.

	LT4 supplementation (n=12)	No LT4 supplementation (n=44)	p*
Age, years	8.3 ± 3.26	9.8 ± 3.23	0.177
Sex (%): Feminine, Masculine	33.3%, 66.7%	45.5%, 54.5%	0.525
Pubertal state: Prepubertal, Pubertal	85.7%, 14.3%	77.8%, 22.2%	1.000
Weight SDS	-1.7 ± 1.30	-1.9 ± 1.11	0.715
Height SDS	-2.6 [-3.07 to -2.29]	-2.6 [-3.14 to -2.36]	0.792
BMI SDS	-0.1 ± 0.87	-0.1 ± 1.21	0.864
GVSDS	-2.2 ± 1.70	-2.1 ± 1.91	0.904
IGF-1 SDS	-1.1 ± 1.31	-1.2 ± 1.24	0.866
FT4 (ng/dL)	1.0 ± 0.07	1.1 ± 0.14	0.002
TSH (µU/L)	2.8 ± 1.23	2.5 ± 0.99	0.312
GH lower peak (ng/mL)	3.5 ± 2.24	3.3 ± 1.79	0.726
rhGH dose (mg/kg/day)	0.030 ± 0.002	0.032 ± 0.004	0.042

Variation of auxological and biochemical parameters in the first two years of rhGH treatment also did not associate with hypothyroidism (Ht SDS variation 1.14 [0.68-1.2] vs 0.91 [0.61-1.61], p=0.841; GV SDS variation 3.3 ± 1.24 vs 4.4 ± 5.61, p=0.785; IGF-1 SDS variation 2.2 ± 1.43 vs 2.5 ± 1.57, p=0.729; FT4 variation -0.1 ± 0.19 vs -0.1 ± 0.18 ng/dL, p=0.869; TSH variation -1.0 ± 1.86 vs -0.8 ± 1.13 µU/L, p=0.737).

Final height SDS of patients who finished rhGH treatment at the end of follow-up (n=35, n=7/12 of those treated with LT4) was -1.16 (-1.73 to -0.82), and it was not different in those with supplemented hypothyroidism (-1.24 [-1.52 to -1.10] vs -1.13 [-1.78 to -0.74], p=1.000). There was a slight difference between final height SDS and PAH SDS globally (n=32, -1.24 [-1.72 to -0.84] vs -0.90 [-1.36 to -0.56]; p=0.047), that was not verified when evaluating separately the group of children treated with LT4 (n=7; final Ht SDS -1.16 [-1.31 to -1.10] vs PAH -1.00 [-1.42 to -0.48]; p=0.398) and the group not treated (n=25; final Ht SDS -1.46 [-1.83 to -0.78] vs PAH SDS -0.88 [-1.35 to -0.56]; p=0.074).

## Discussion

In this retrospective cohort study of children with isolated idiopathic GHD, we evaluated the effects of rhGH during the first two years of treatment, particularly on thyroid function. As expected, treatment with rhGH improved patients’ Wt SDS, height SDS, GV SDS, and IGF-1 SDS.

Twelve patients (21.4%) with isolated idiopathic GHD developed clinical hypothyroidism during rhGH therapy, starting replacement with LT4 at 0.7 (0.58-1.44) mcg/kg/day. The timing of hypothyroidism development was highly variable, and it seemed to develop earlier in patients with lower FT4 levels at baseline. Although the timing of hypothyroidism did not associate with somatropin doses, relevant thyroid function changes were associated with a better response to treatment. Other authors previously reported the need for LT4 during rhGH therapy [6, 15-18], that occurred earlier in the course of treatment [25], but only two studies directly evaluated patients with isolated idiopathic GHD [15, 18]. Researchers suggest that clinically relevant hypothyroidism mostly arises in patients with MPHD, organic lesions on MRI or genetic defects causing GHD. Seminara et al. [26], who evaluated a group of patients similar to ours, reported that decreases in total and FT4 concentrations and increases in total and FT3 were transient and disappeared during further observation. However, most studies reported thyroid function outcomes from one to four years of follow-up [2, 12, 17]. Through longer follow-up of patients’ thyroid function, we showed that LT4 requirement may occur at variable times during rhGH therapy, and that it is an important outcome in patients with isolated idiopathic GHD.

LT4 requirement was temporary in our patients. All of the patients who completed treatment with rhGH except one retrieved to baseline FT4 levels (after LT4 suspension). TSH levels were still lower after 6-12 months of rhGH interruption, but this could be explained by a slower recuperation of TSH comparing to FT4.

As such, in patients with isolated idiopathic GHD, there appears to be a clear causal relationship between prolonged rhGH therapy and clinical hypothyroidism, that reverts to euthyroidism on cessation of therapy [15, 18].

Our analysis of thyroid function evolution during rhGH therapy in patients that were not under LT4, showed a reduction in FT4 and TSH levels that was sustained for the two years of assessment. As stated, several studies reported controversial results on the effect of rhGH on thyroid function [2-7, 15, 27]; some authors reported significant changes in FT4 and T3 in the first 6-12 months of rhGH therapy, with the posterior return to normal levels [17, 20, 26]. TSH levels did not vary significantly in most studies [15, 18, 26], although one study reported an initial increase, with return to baseline levels in the second year of therapy [18]. These results are distant from ours. Many factors can contribute to the discrepancy in results, but the heterogeneity of the patients included might explain the major differences, as the expected impact of rhGH in different patients is not the same.

Previously it was demonstrated that this extra-thyroidal conversion of T4 to T3 also works at the level of central nervous system, as FT4 concentrations in the cerebrospinal fluid significantly decreased during rhGH therapy with a probable beneficial impact on mood and behavior [20].

As we have excluded patients with genetic defects, organic lesions, or other changes in MRI that could justify a hypopituitarism, unmasking previously unrecognised central hypothyroidism as part of combined pituitary hormone deficiencies was not the cause for thyroid function changes in this setting [2, 17, 28, 29]. The direct effect of rhGH on hypothalamic thyrotropin-releasing hormone (TRH) response, or inhibition of the TSH responses to TRH could be underlying mechanisms in these patients [30], or an indirect effect of GH mediated by an increase in the hypothalamic somatostatinergic tone. Indeed it is well recognized that somatostatin is a factor inhibiting TSH secretion.

To our knowledge, this is the first study exploring predictive factors for hypothyroidism during rhGH therapy in a subset of children with isolated idiopathic GHD. Unadjusted comparison of patients who were treated with LT4 with those who were not, showed a slightly lower rhGH dose in the first group, which might be in line with the hypothesis that best responders to rhGH are also more prone to thyroid hormone variations; this is also in agreement with Wong et al. [25], who evaluated patients with multiple GHD etiologies and reported lower doses of rhGH in patients who developed hypothyroidism.

Portes et al. [2] referred to severe GHD as a state of deficient conversion of T4 to T3, leading to increased serum FT4 and rT3, and decreased serum T3, all corrected by GH replacement. We evaluated the impact of GHD severity in the need of LT4, but the lower GH peak of two provocative tests did not associate with the appearance of clinically relevant hypothyroidism. However, Wong et al. [25], who studied 119 patients treated with rhGH for multiple reasons, reported an association between GHD severity and the development of hypothyroidism.

We also found that lower FT4 levels at baseline (adjusted for patient’s age), might be predictive of the urge of hypothyroidism during therapy with rhGH. Further research is needed to understand why these patients develop significant thyroid function changes, being that variation of biochemical parameters per se, on the other hand, does not appear to be determinant for the emergence of hypothyroidism.

The final height SDS of patients with properly treated hypothyroidism was similar to their counterparts without hypothyroidism, and both groups achieved a final height SDS that was not different from PAH SDS. Giavoli et al. [12] reported a decline in GV before the start of LT4 supplementation and achievement of euthyroidism in patients with MPHD, Lippe et al. [6] documented the need of LT4 to achieve maximal growth rate, and Wong et al. [25] found decreased growth velocity SDS in patients with hypothyroidism. This evidence supports that adequate thyroid hormone replacement enhances the rhGH effectiveness in children with GH deficiency. Even if initial reduction in FT4 levels is a result of enhanced peripheral conversion to T3 at first, we found that this reduction is prolonged and might exacerbate with time, eventually impairing children’s growth if not appropriately corrected [12].

Our study is one of the largest in a pediatric population with isolated idiopathic GHD. Being the most common cause of GHD in children, further knowledge of how rhGH therapy impacts thyroid hormones in these patients is of great importance. A better understanding of predisposing factors to clinically relevant hypothyroidism in children treated with rhGH will help recognize patients in need for tighter and more frequent assessment of thyroid function during long-term therapy. Awareness for the occurrence of transient hypothyroidism, not only in children with MPHD, particularly in those with lower pretreatment FT4 levels, might be crucial in optimizing the patient’s final height.

The main limitation of our study is its retrospective design. As the measurement of T3 or FT3 levels is not routinely performed in euthyroid or hypothyroid patients, most of our patients did not have T3 levels measured, resulting in absence of analyses of FT3 and rT3, in contrary to other studies. Understanding if T3 levels follow the decrease in FT4 will help understand the true significance of the observed changes in thyroid function of our patients. Different follow-up periods within patients, since not all of them finished rhGH treatment at the time of the study, can also limit our conclusions, but an attempt to standardize follow-up time would drastically reduce our sample and have even more impact on generalization of results. This can also underestimate the prevalence of hypothyroidism in these patients, as some of them may still develop the need for LT4 during the course of treatment. One limitation is that due to sample size (the group of LT4 treated patients had only 12 patients) and risk of undermining the power of the analyses, we were unable to perform multiple regression and adjust for potential confounders, which limits the extent of our conclusions.

## Conclusions

FT4 and TSH levels decrease significantly during rhGH therapy in children with isolated idiopathic GHD. Patients with lower baseline FT4 levels might be at risk of transient hypothyroidism, which can occur at any time during GH treatment. Adequate LT4 supplementation allows patients to achieve PAH, and a final height that is not different from those who don’t need hormonal replacement.
